# Supplementary material for: Expression of tumor antigens within an oncolytic virus enhances the anti-tumor T cell response
Source: Nat Commun. 2024 Jun 27;15:5442. doi: 10.1038/s41467-024-49286-x (PMC11211353; doi:10.1038/s41467-024-49286-x)
Supplement: Supplementary file 1 — Supplementary Information [file 41467_2024_49286_MOESM1_ESM.pdf]

Expression of Tumor Antigens within an Oncolytic Virus enhances the Anti-Tumor T Cell Response

Supplementary Information

Mason J. Webb[1,2], Thanich Sangsuwannukul[2], Jacob van Vloten[2], Laura Evgin[2,3,4], Benjamin Kendall[2], Jason Tonne[2], Jill Thompson[2], Muriel Metko[2], Madelyn Moore[2,5], Maria P. Chiriboga Yerovi[2], Michael Olin[6], Antonella Borgatti[7,8,9], Mark McNiven[10], Satdarshan P. S. Monga[11], Mitesh J. Borad[12], Alan Melcher[13], Lewis R. Roberts[14], Richard Vile[2,15,16*]

[1]Department of Hematology/Medical Oncology, Mayo Clinic, Rochester, MN 55905, USA; [2]Department of Molecular Medicine, Mayo Clinic, Rochester, MN 55905, USA; [3]Department of Medical Genetics, University of British Columbia, Vancouver, BC V5Z1L3, Canada; [4]Michael Smith Genome Sciences Department, BC Cancer Research Institute, Vancouver, BC V5Z1L3, Canada; [5]Department of Pharmacology, University of Minnesota, Minneapolis, MN 55455, USA; [6]Division of Pediatric Hematology and Oncology, University of Minnesota, Minneapolis, MN 55455, USA; [7]Department of Veterinary Clinical Sciences, University of Minnesota, St. Paul, MN 55108; [8]Masonic Cancer Center, University of Minnesota, Minneapolis, MN 55455; [9]Clinical Investigation Center, University of Minnesota, St. Paul, MN 55108 ; [10]Mayo Center for Biomedical Discovery, Mayo Clinic, Rochester, MN 55905, USA; [11]Pittsburgh Liver Institute, University of Pittsburgh and UPMC, Pittsburgh, PA 15261, USA; [12]Department of Hematology/Medical Oncology, Mayo Clinic, Phoenix, AZ 85054, USA; [13]Division of Radiotherapy and Imaging, Institute of Cancer Research, Chester Beatty Laboratories, London SW3 6JB, UK; [14]Department of Gastroenterology and Hepatology, Mayo Clinic, Rochester, MN 55905, USA; [15]Department of Immunology, Mayo Clinic, Rochester, MN 55905, USA; [16]Joan Reece Department of Immuno-oncology, King's College London, London, UK'

[*]Correspondence should be addressed to Richard G. Vile, Ph.D.

Mayo Clinic, Guggenheim 18, 200 1st St SW, Rochester, MN 55905

Phone: 507-284-3178 / FAX: 507-266-2122 / vile.richard@mayo.edu

Fig.S1A

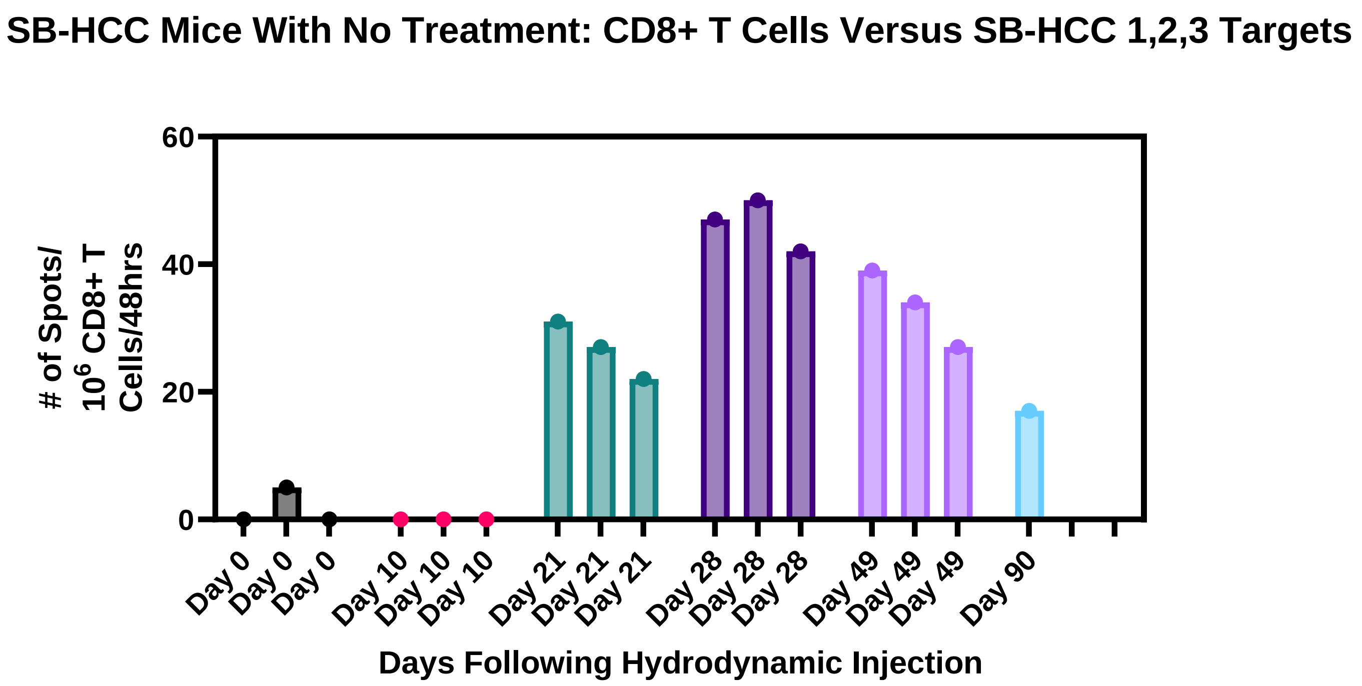

Fig.S1.B

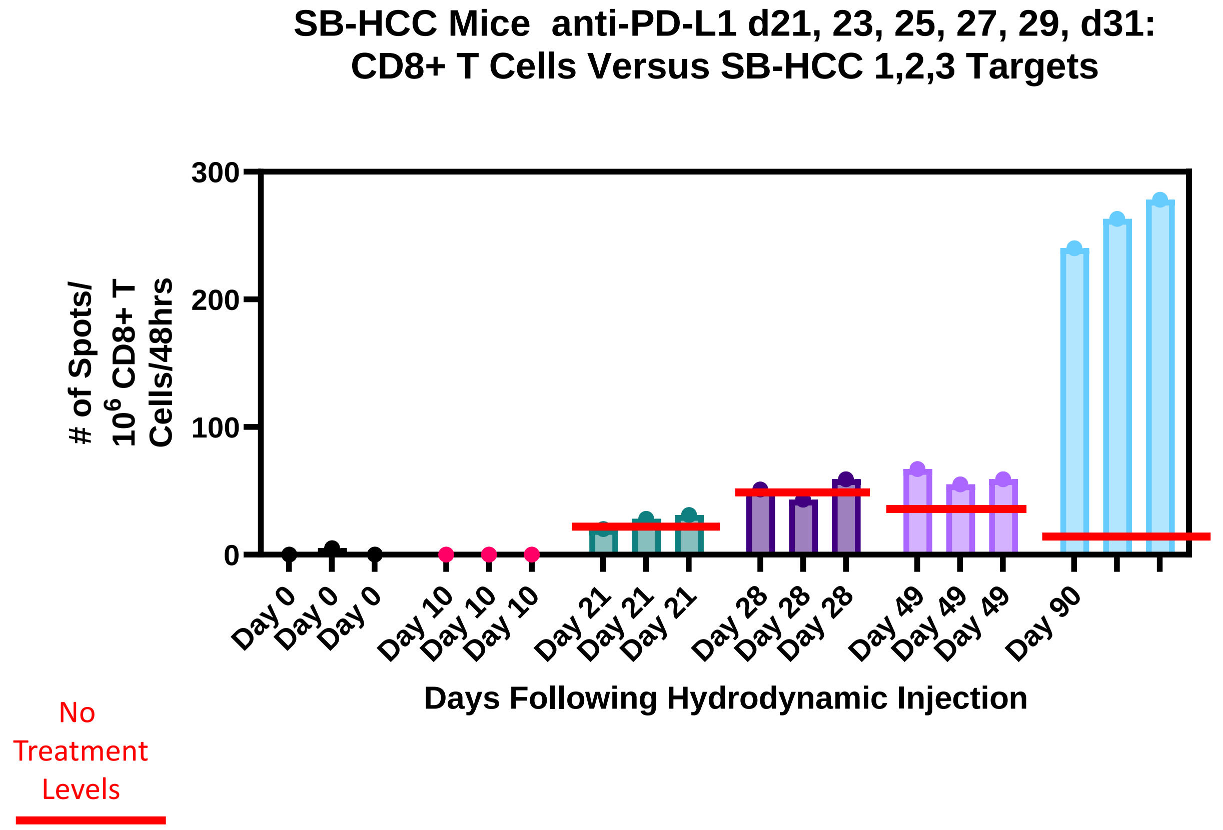

Supplemental Figure 1. Anti-PD-L1 ICB Maintains the Anti-Tumor T Cell Response From d28-d49 And Then Significantly Expands It Through Day 90.

Following hydrodynamic injection of hMet + S45Y ß-Catenin (day 0), animals were left untreated (**A**) or were treated starting on days 21,23,25,28,30,32 with anti-PD-L1 (200µg/injection) (**B**). Spleens from 3 mice per group per timepoint were harvested at the day of hydrodynamic injection (d0) or on days 10, 21, 28, 49 and, if still surviving, day 90 post hydrodynamic injection (only 1 mouse in **A.** at d90). CD8+ T cells were purified from the splenocytes and co-cultured with a 1:1;1 mixture of live SB-HCC 1,2,3 explant cells as targets at an effector:target ratio of 10:1 in IFNγ ELISpot plates (R&D Systems Murine IFN-gamma ELISpot kit EL485). Plates were developed 48 hrs later and the number of spots was counted.

Fig.S2.A

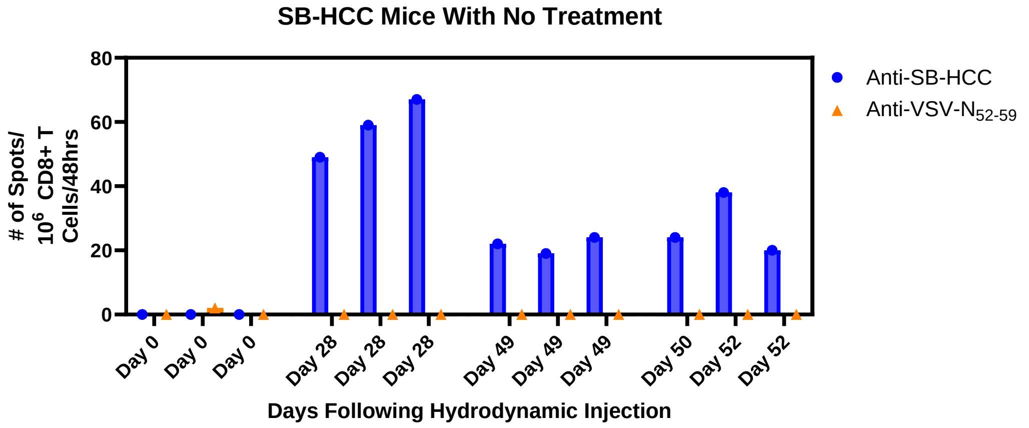

Fig.S2.B

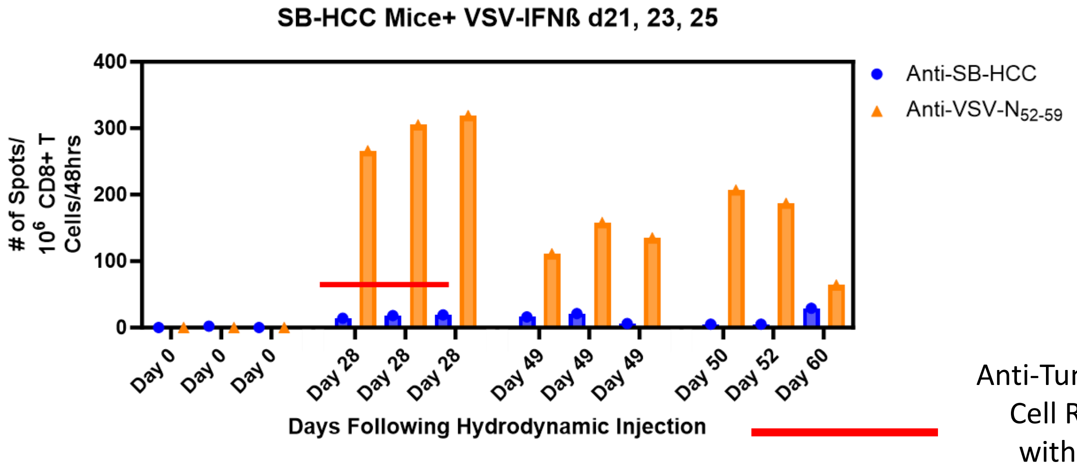

Anti-Tumor CD8+ T Cell Response with No VSV Treatment

Fig.S2.C

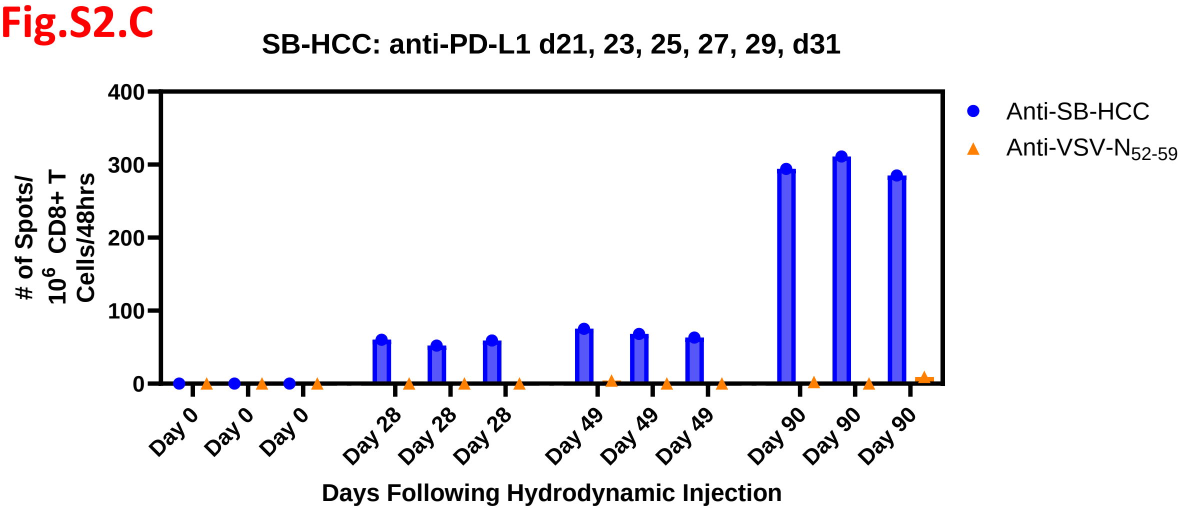

Fig.S2.D

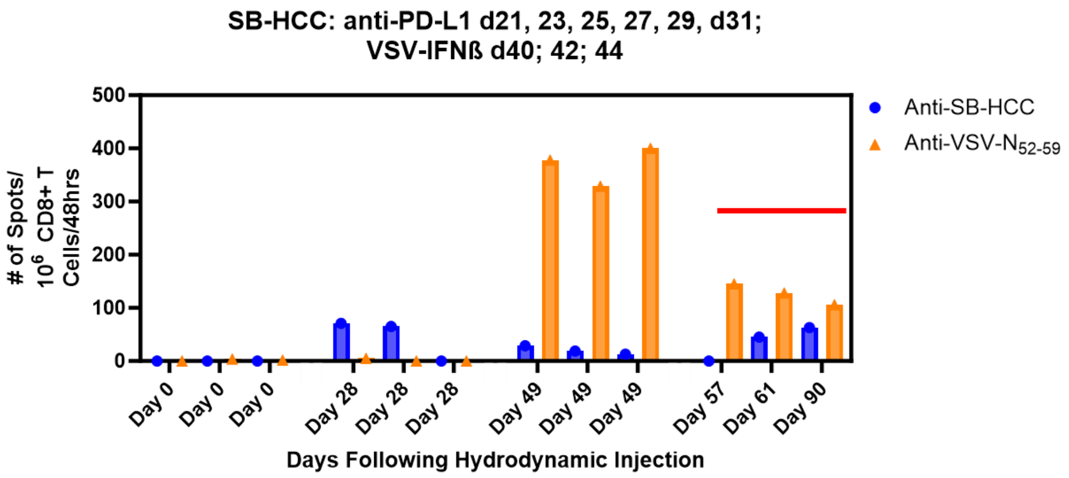

Anti-Tumor CD8+ T Cell Response with anti-PD-L1 Treatment Alone

Supplemental Figure 2. Treatment with VSV-IFNß Significantly Diminishes The Strength of the anti-PD-L1-Enhanced Anti-Tumor CD8+ T Cell Response And Replaces It With A Potent Anti-VSV CD8+T Cell Response.

Following hydrodynamic injection of hMet + S45Y ß-Catenin (day 0), animals were left untreated (**A**); or were treated with 10^7 pfu of VSV-IFNß on days 21,23,25 (**B**); or with anti-PD-L1 (200µg/injection) on days 21,23,25,28,30,32 (**C**); or with anti-PD-L1 (200µg/injection) on days 21,23,25,28,30,32 and with VSV-IFNß on days 38,40,42 (**D**). Spleens from 3 mice per group per timepoint were harvested at the day of hydrodynamic injection (d0) or on days 10, 28, 49 and then when mice succumbed to disease or at day 90 if they survived. CD8+ T cells were purified from the splenocytes and co-cultured with a 1:1;1 mixture of live SB-HCC 1,2,3 explant cells as targets at an effector:target ratio of 10:1 or with 5µg/ml of the immunodominant VSV-N_{52-59} peptide to stimulate a recall response to VSV (anti-VSV-N_{52-59} CD8+ T cell response) in IFNγ ELISpot plates. Plates were developed 48 hrs later and the number of spots was counted.

Fig.S3

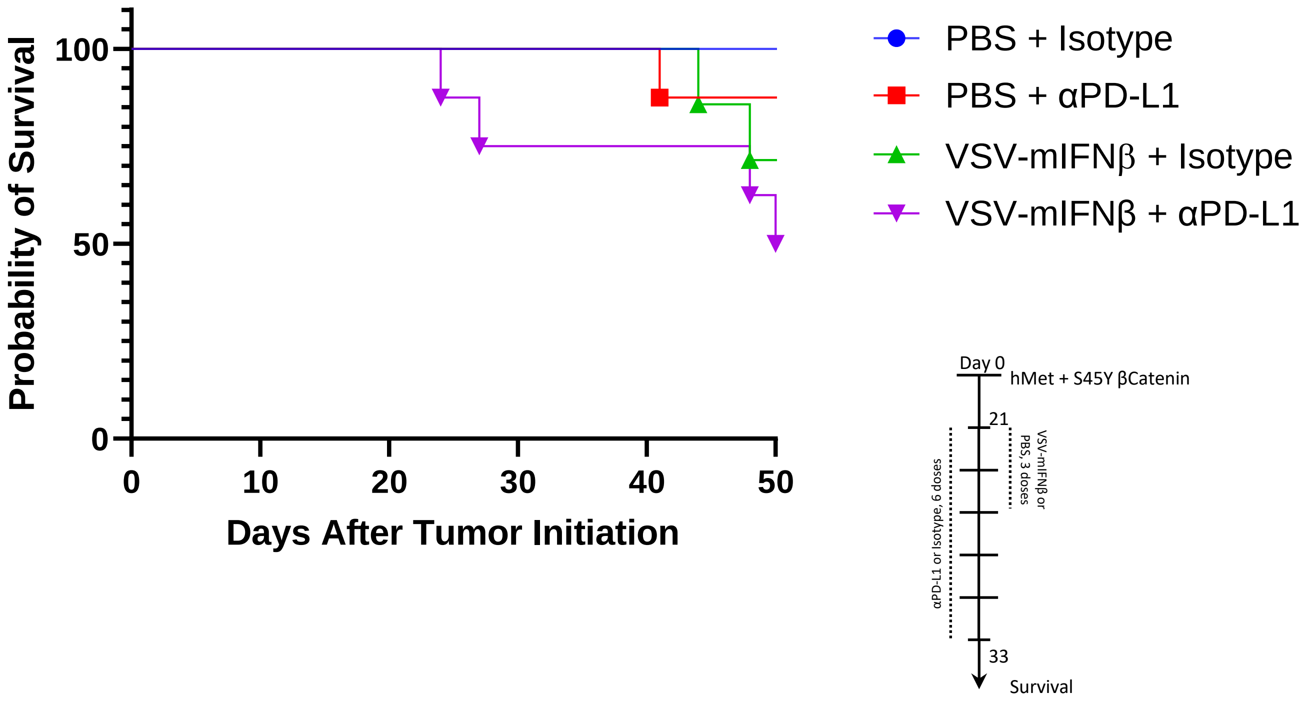
Probability of Survival
100
50
0
0
10
20
30
40
50
Days After Tumor Initiation
PBS + Isotype
PBS + αPD-L1
VSV-mIFNβ + Isotype
VSV-mIFNβ + αPD-L1
Day 0
hMet + S45Y βCatenin
21
VSV-mIFNβ or PBS, 3 doses
αPD-L1 or Isotype, 6 doses
33
Survival

Supplemental Figure 3. Concomitant ICI with VSV-IFNß.

Mice hydrodynamically injected with hMet + S45Y ß-Catenin at day 0 were treated with control IgG isotype or anti-PD-L1 on days 21, 23, 25, 28, 30, 33 (6 doses, 200µg per dose) and with PBS or VSV-mIFNß on days 21, 23 and 25 (3 doses, 10^8 pfu/dose). Survival with time is shown.

Fig.S4A

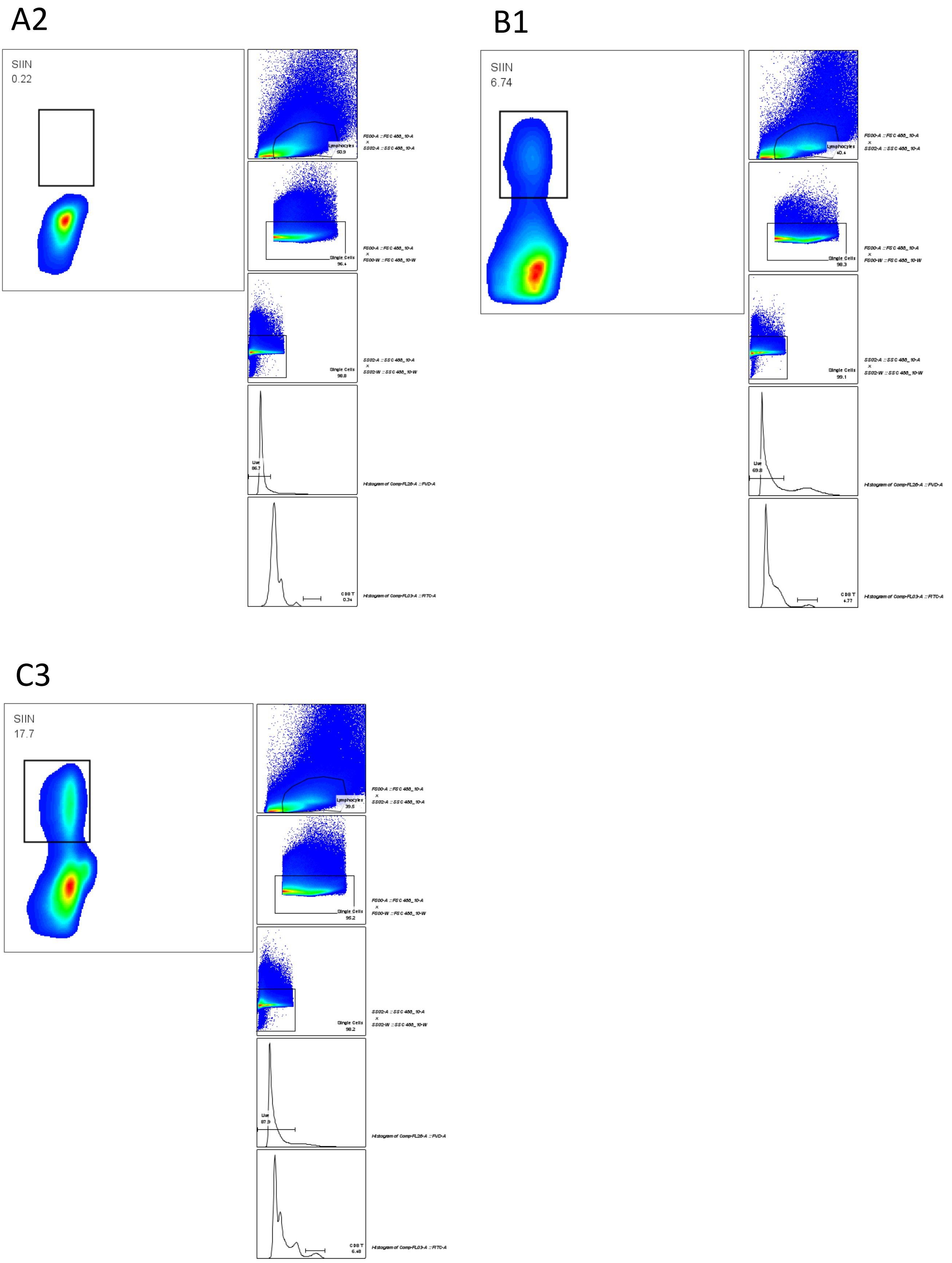

Fig.S4B

A3

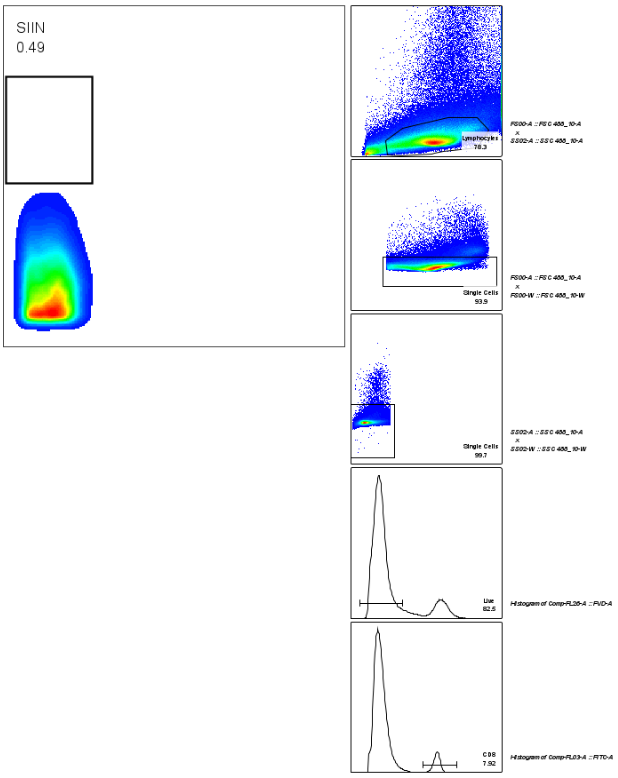

B4

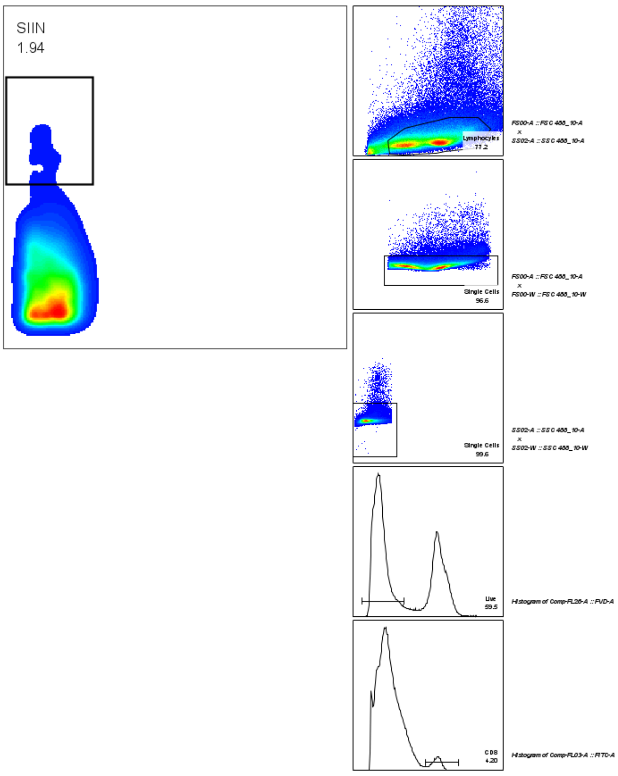

C3

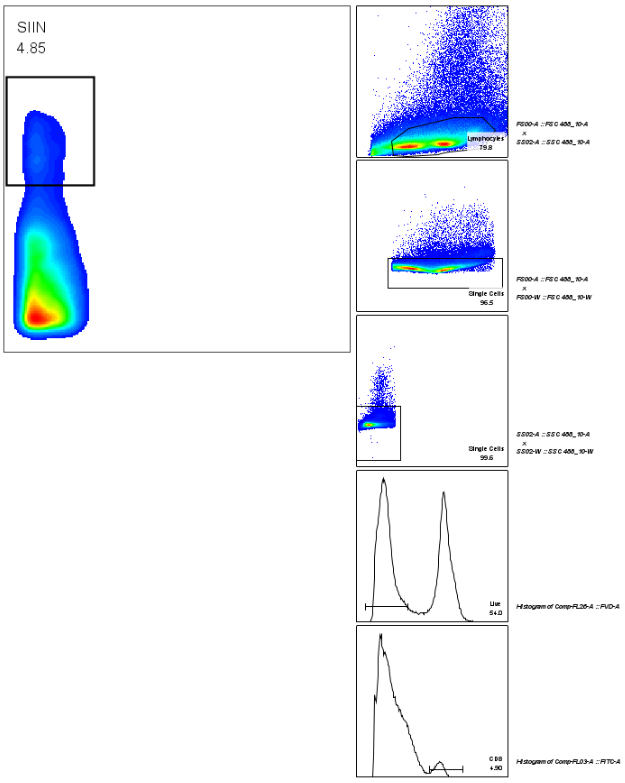

Fig.S4C

A6

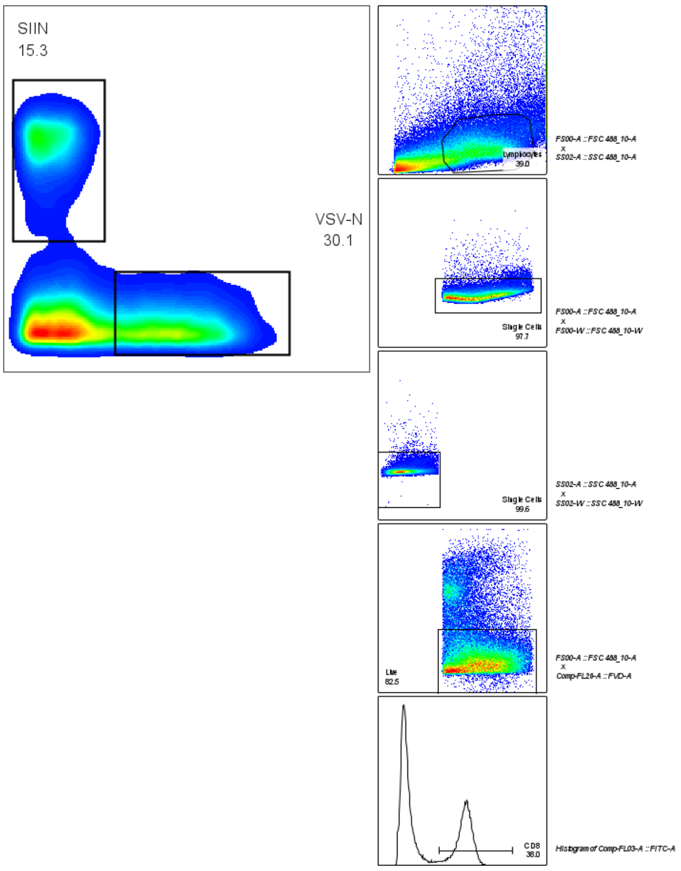

B1

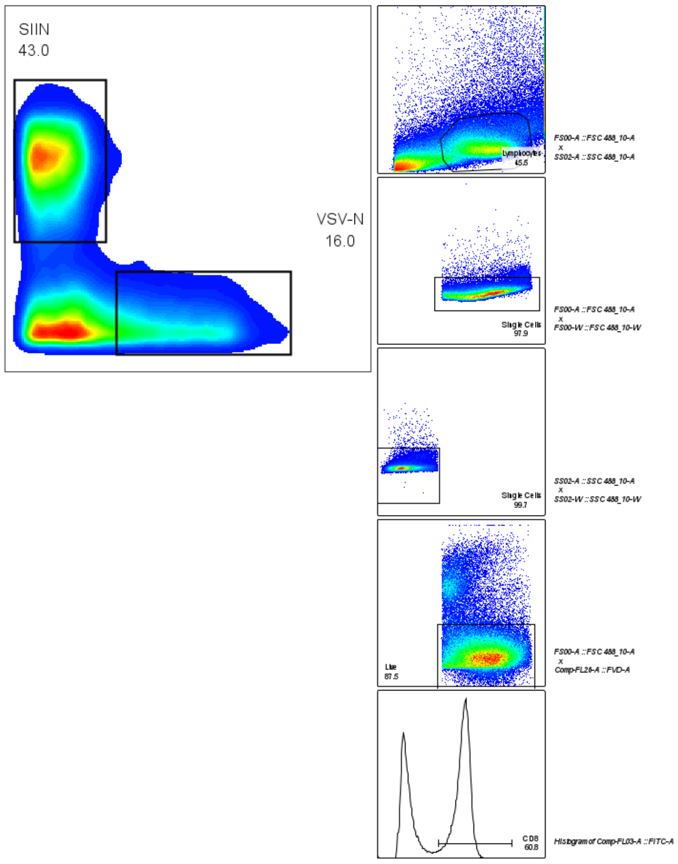

Fig.S4D

A4

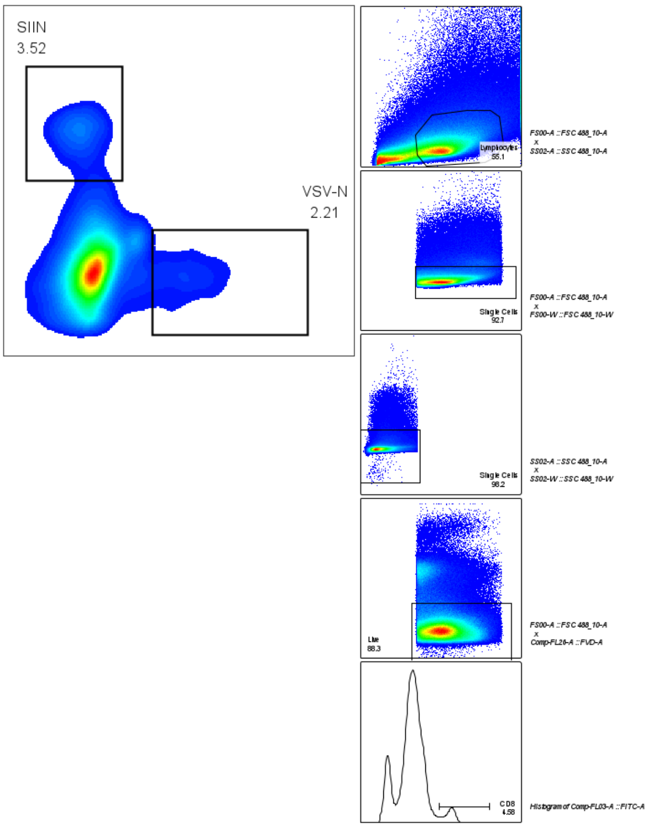
SIIN
3.52
VSV-N
2.21
Lymphocytes
55.1
FS00-A :: FSC 488_10-A
x
SS02-A :: SSC 488_10-A
Single Cells
92.7
FS00-A :: FSC 488_10-A
x
FS00-W :: FSC 488_10-W
Single Cells
98.2
SS02-A :: SSC 488_10-A
x
SS02-W :: SSC 488_10-W
Live
88.3
FS00-A :: FSC 488_10-A
x
Comp-FL26-A :: FVD-A
CD8
4.58
Histogram of Comp-FL03-A :: FITC-A

B3

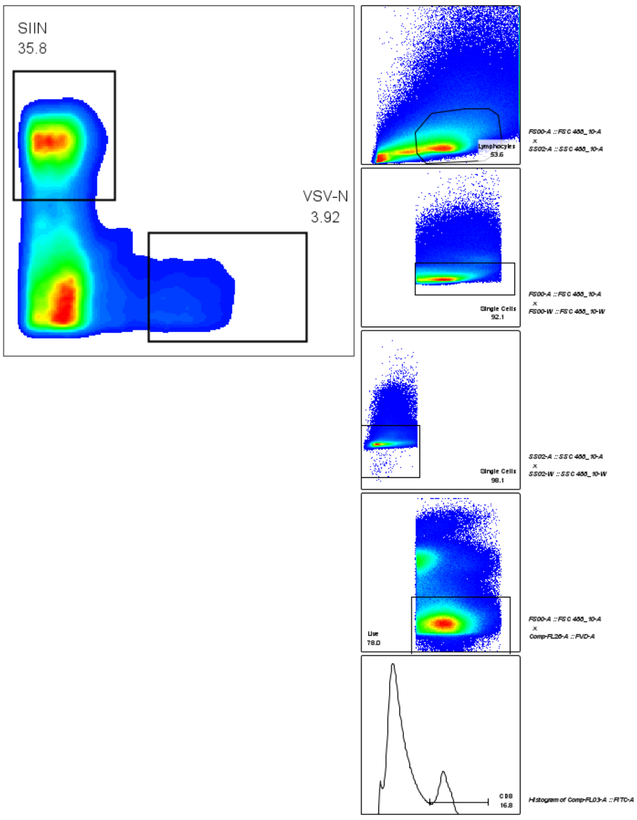
SIIN
35.8
VSV-N
3.92
Lymphocytes
53.6
FS00-A :: FSC 488_10-A
x
SS02-A :: SSC 488_10-A
Single Cells
92.1
FS00-A :: FSC 488_10-A
x
FS00-W :: FSC 488_10-W
Single Cells
98.1
SS02-A :: SSC 488_10-A
x
SS02-W :: SSC 488_10-W
Live
78.0
FS00-A :: FSC 488_10-A
x
Comp-FL26-A :: FVD-A
CD8
16.8
Histogram of Comp-FL03-A :: FITC-A

Supplemental Figure 4. Gating strategy for Figure 4 flow cytometry. For all gating (**A-D**), lymphocytes from either liver or spleen were gated by size and isolated to single cells. Live cells were selected using staining with fixable live dead viability dye. CD8 cells were selected using a CD8-FITC antibody, then gated with SIIN-APC and VSV NP52–59 RGYVYQGL-Brilliant Violet 421. Each labeled gating lineage represents a single animal. (**A**) corresponds to **Figure 4A**, (**B**) to **4B**, (**C**) to **4G**, and (**D**) to **4H**.

Fig.S5A

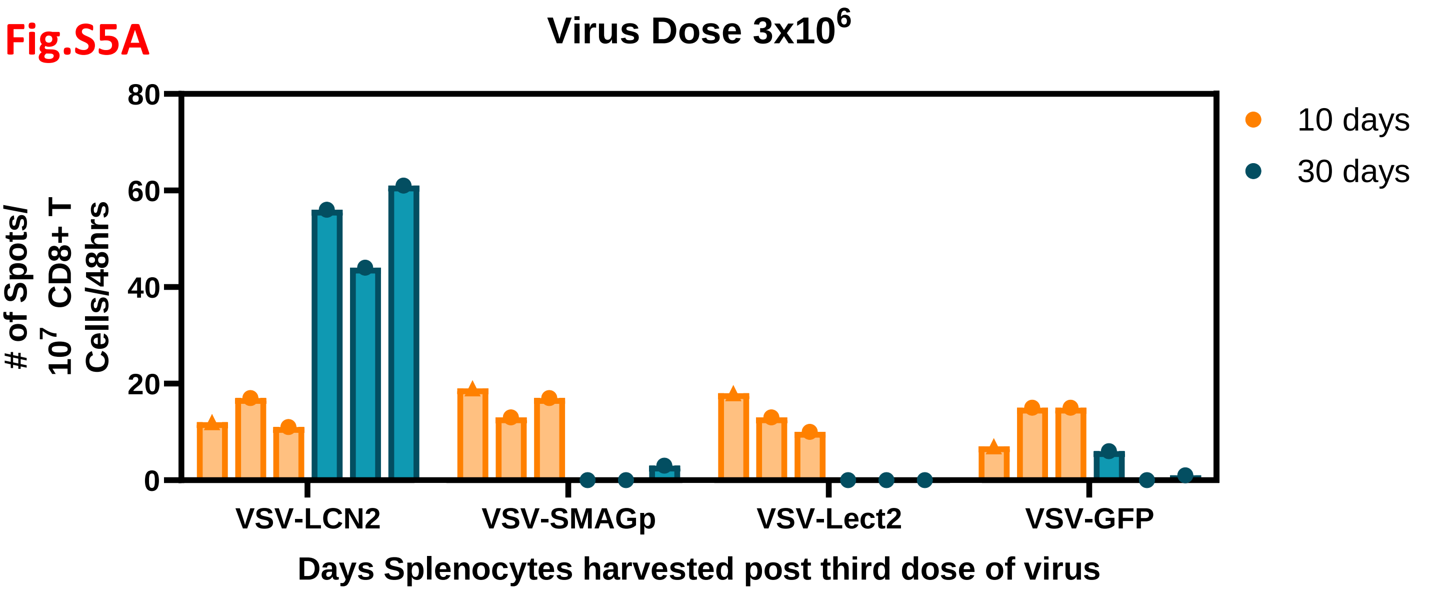

Fig.S5B

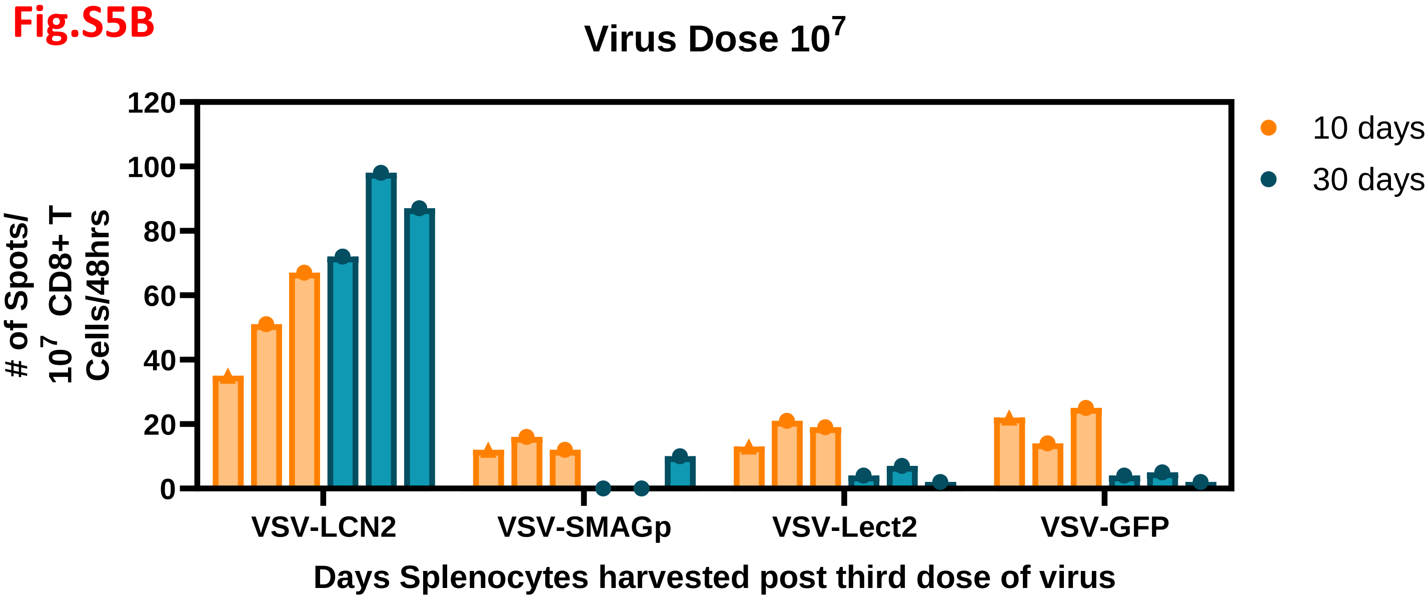

Supplemental Figure 5. A weak, slow developing anti-LCN-2/HCC CD8+ T cell response develops following low dose ($3x10^6$ pfu) VSV-*Lcn2* treatment while neither SMAGp nor Lect2 are immunogenic in this model.

Following hydrodynamic injection of hMet + S45Y ß-Catenin (day 0) mice were treated with anti-PD-L1 (200µg/injection; days 5,7,9,12,14,16) followed by (**A**) $3x10^6$ pfu or (**B**) 10^7pfu of VSV-IFNß-*Lcn2;* VSV-IFNß-*Smagp;* VSV-IFNß-*Lect2;* or VSV-GFP on day 21,22,23. 10 (d33) or 30 (d63) days later spleens from 3 mice per group per timepoint were harvested. CD8+ T cells were purified from the splenocytes and co-cultured with a 1:1;1 mixture of live SB-HCC 1,2,3 explant cells as targets at an effector:target ratio of 10:1 in IFNγ ELISpot plates. Plates were developed 48 hrs later and the number of spots was counted.

Fig.S6A

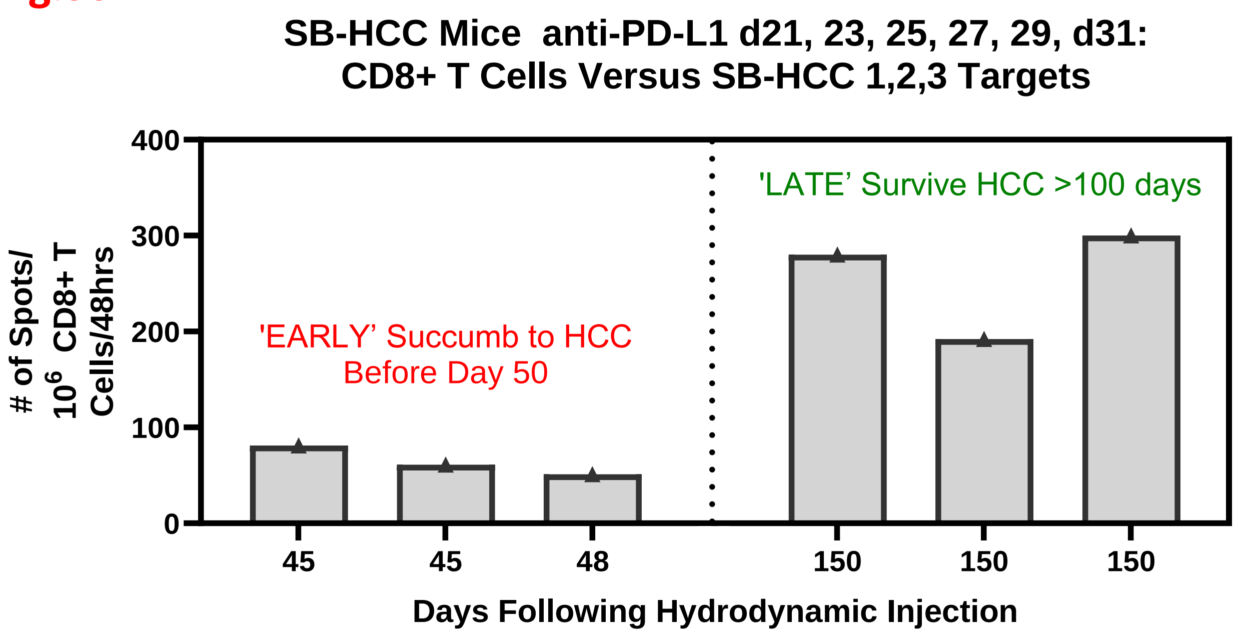

Fig.S6B

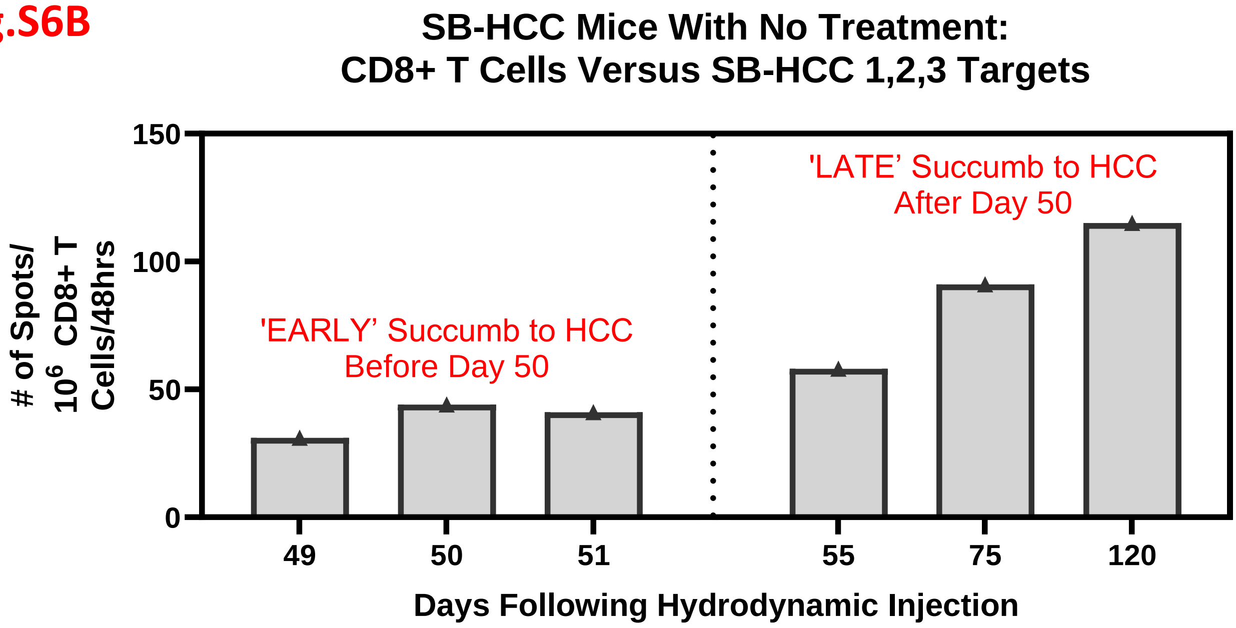

Fig.S6C

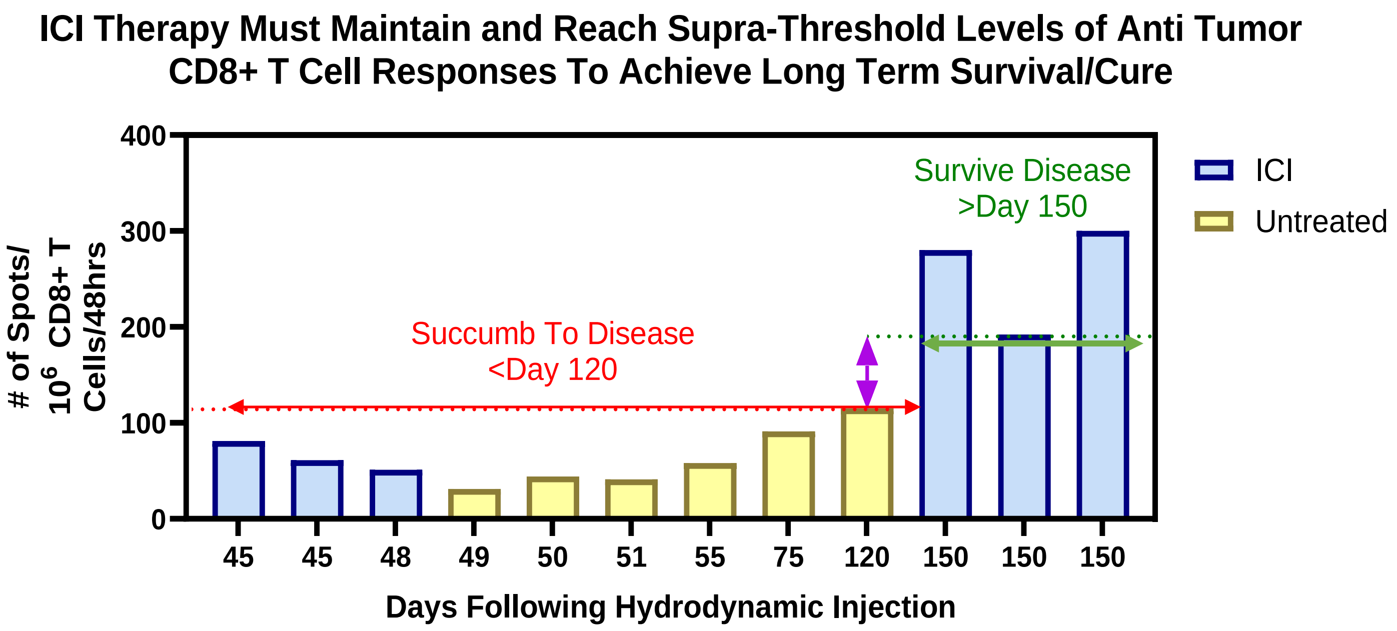

Successful Treatment of HCC Anti-PD-L1 Treatment Must Maintain/Reach a Supra-Threshold Level of CD8+ Anti Tumor T Cell Reactivity (>~115-~200IFNg Spots/10^6 CD8+ T cells)

Supplemental Figure 6. Successful anti-PD-L1 treatment must achieve a supra-threshold level of CD8+ anti-tumor T cell reactivity.

Following hydrodynamic injection of hMet + S45Y ß-Catenin (day 0), mice were (**A**) treated starting on days 21,23,25,28,30,32 with anti-PD-L1 (200µg/injection) (6/grp). Mice treated with anti-PD-L1 ICI either die early (<50days) at the same rate as untreated mice or they survive significantly longer (≥150days) (‘Cure’). Spleens were harvested from mice which either succumb to disease or survived (>150 days). CD8+ T cells were purified from the splenocytes and co-cultured with a 1:1;1 mixture of live SB-HCC 1,2,3 explant cells as targets at an effector:target ratio of 10:1 in IFNγ ELISpot plates. Plates were developed 48 hrs later and the number of spots was counted. **B.** Following hydrodynamic injection of hMet + S45Y ß-Catenin (day 0), mice were left treated (Control IgG). Spleens were harvested from the mice at the timepoint at which they succumbed to disease (all 6 mice). CD8+ T cells were purified from the splenocytes and co-cultured with a 1:1;1 mixture of live SB-HCC 1,2,3 explant cells as targets at an effector:target ratio of 10:1 in IFNγ ELISpot plates. Plates were developed 48 hrs later and the number of spots was counted. **C.** Combining the data of **A** & **B:** Whether treated with anti-PD-L1 (ICI) or Left Untreated (U), SB-HCC mice either succumb to disease or are ‘cured’ (>d150). Despite the low numbers of data points, there is a trend to a positive correlation between length of survival and detectable levels of CD8+ anti-tumor T cell reactivity. Therefore, for successful treatment of HCC anti-PD-L1 treatment must maintain/achieve a supra-threshold level of CD8+ anti-tumor T cell reactivity (>~115-~200IFNγ Spots/10^6 CD8+ T cells).

Fig. S7A

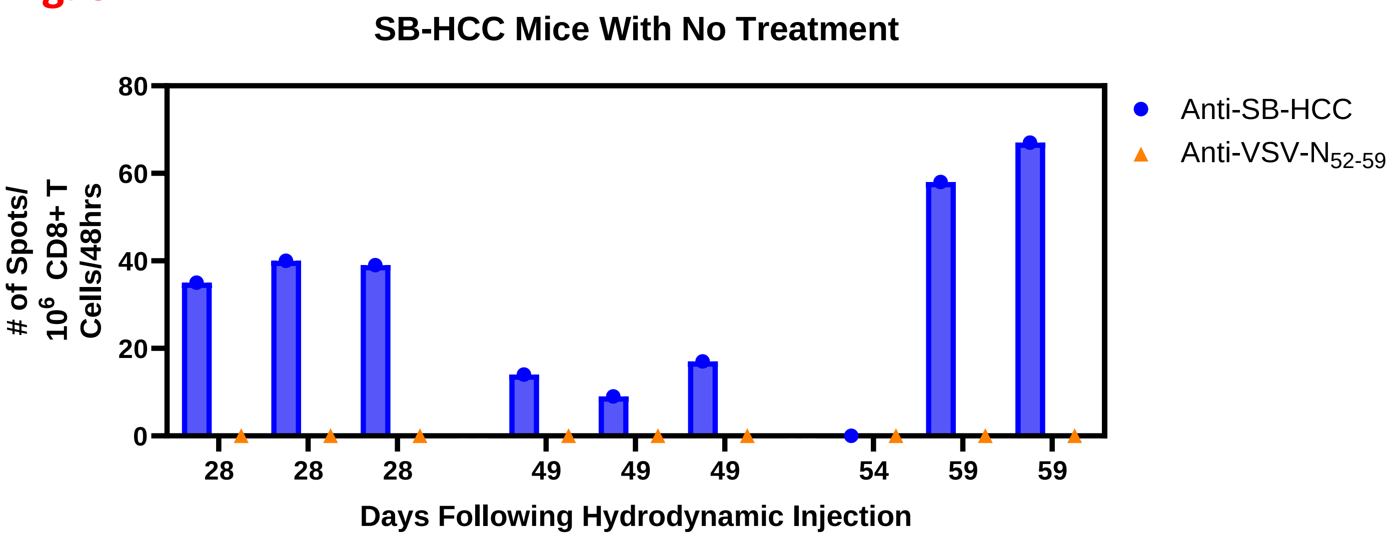

Fig. S7B

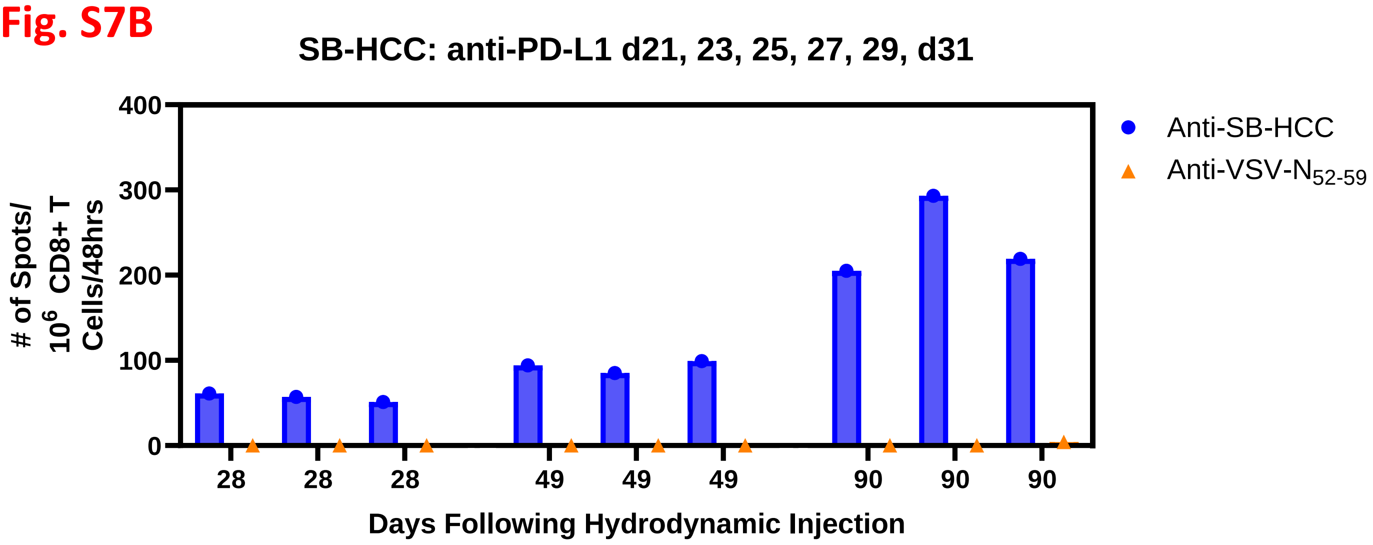

Fig. S7C

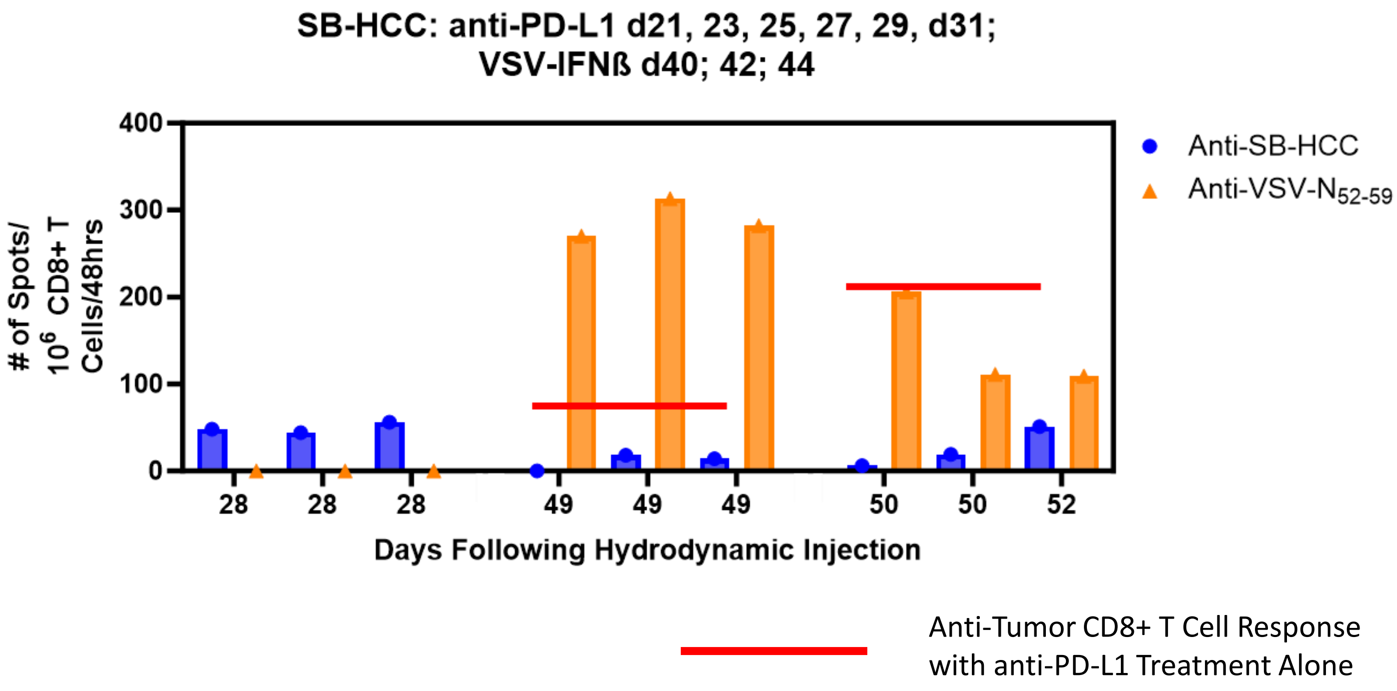

Fig. S7D

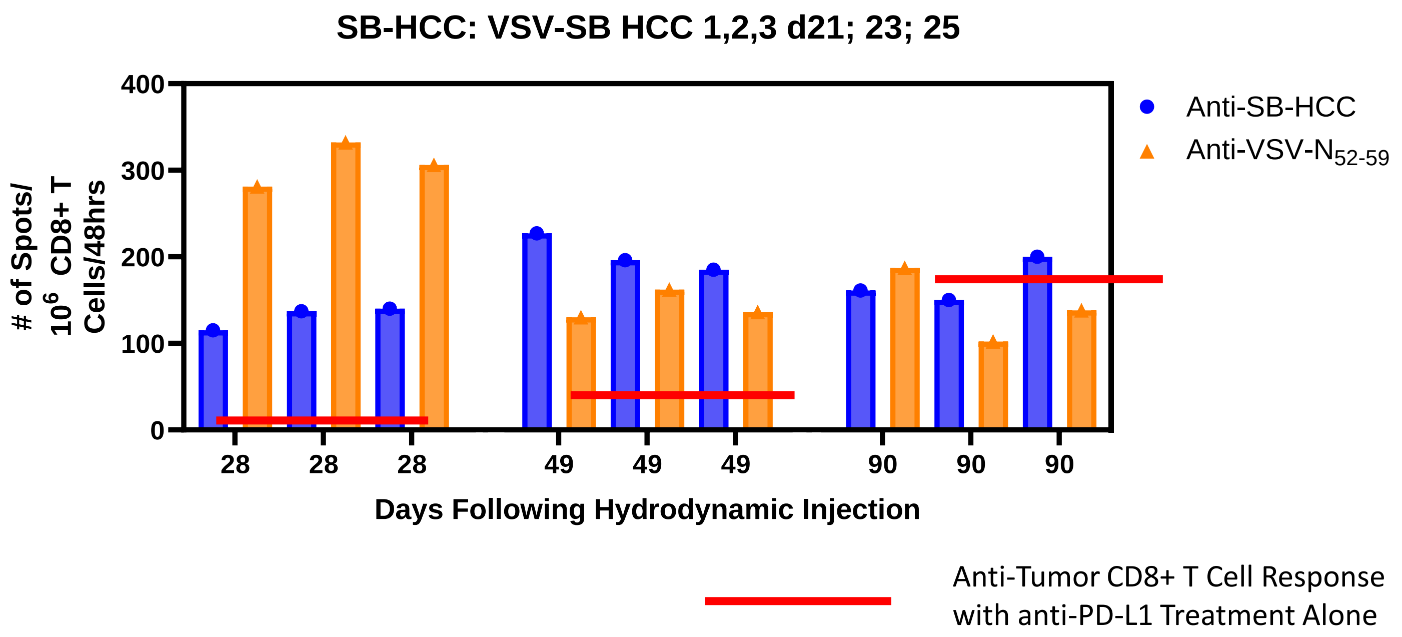

Fig. S7E

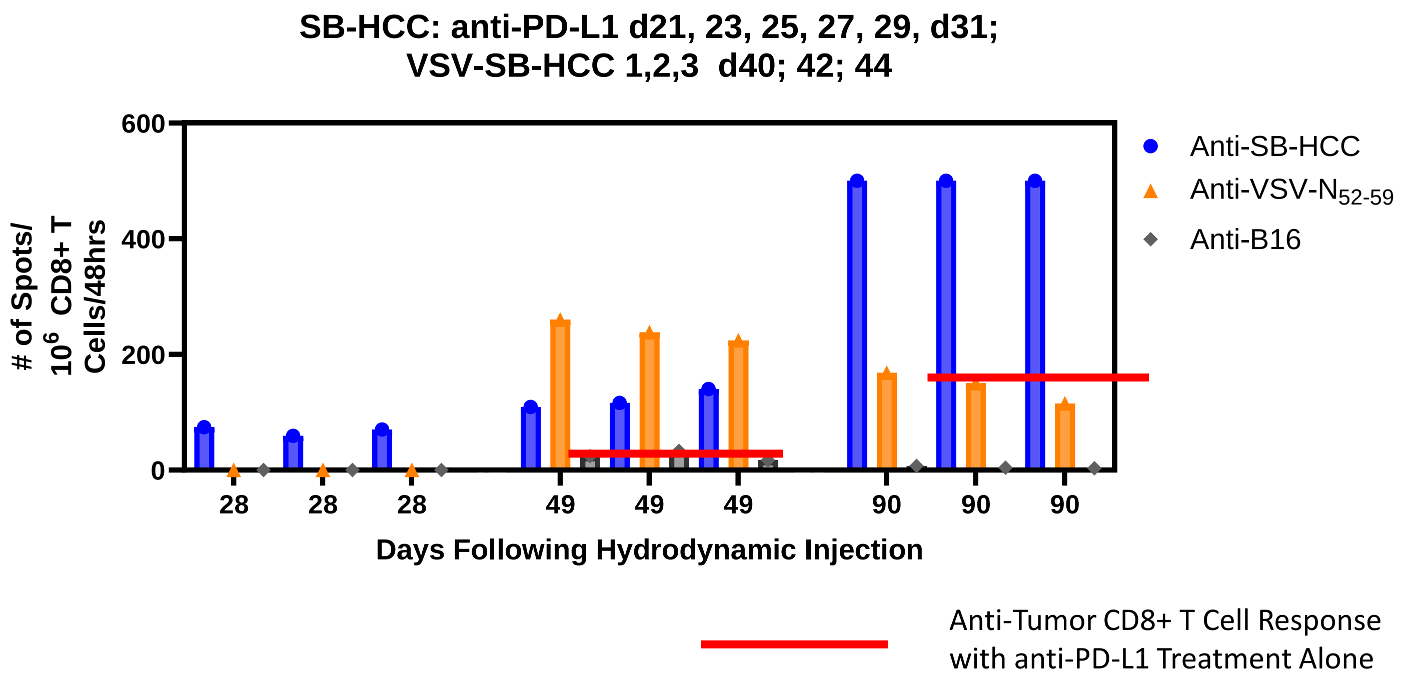

Fig. S7F

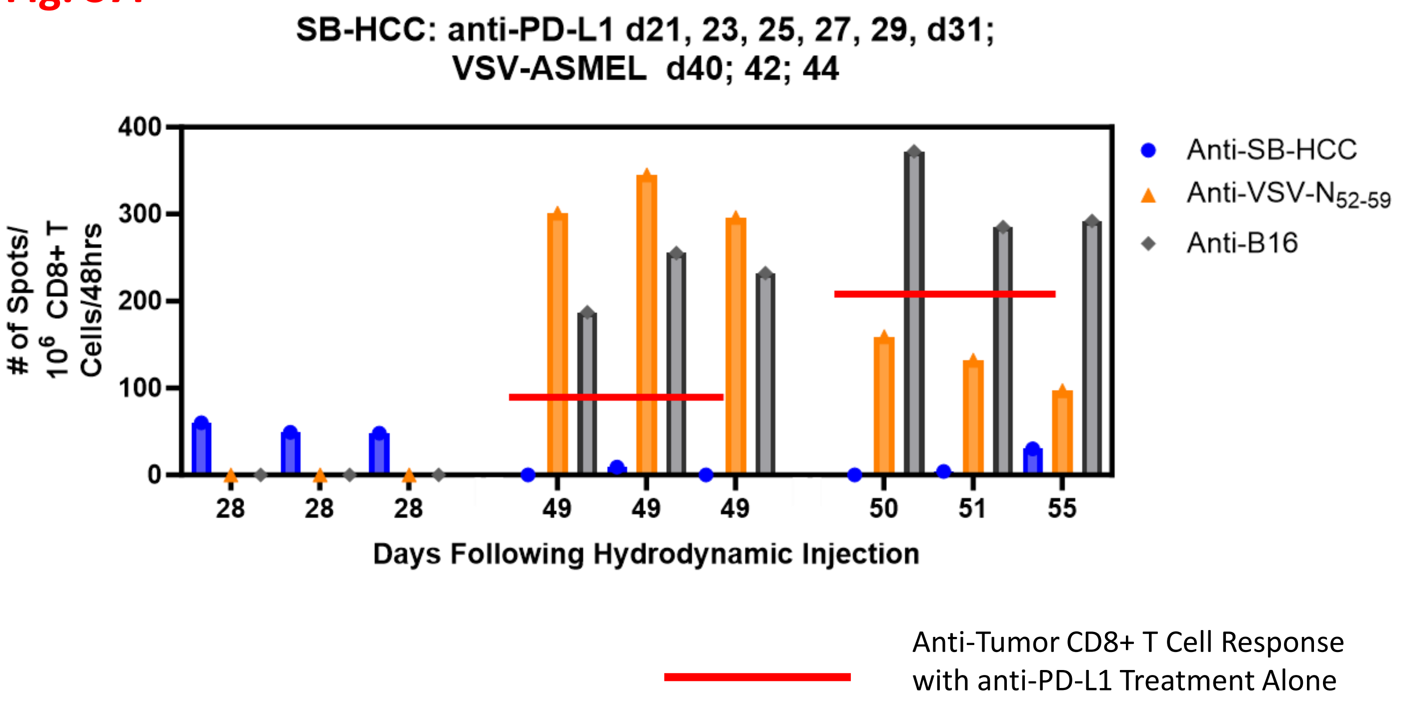

Supplemental Figure 7. Treatment with anti-PD-L1+VSV-IFNß-SB-HCC 1,2,3 significantly enhances the anti-PD-L1-enhanced anti-tumor CD8+ T Cell Response whilst co-existing with the anti-VSV CD8+T Cell Response.

Following hydrodynamic injection of hMet + S45Y ß-Catenin (day 0), animals were left untreated (**A**) or were treated on days 21,23,25,28,30,32 with anti-PD-L1 (200µg/injection) (**B**). CD8+ T cells were purified from the splenocytes and co-cultured with a 1:1;1 mixture of live SB-HCC 1,2,3 explant cells as targets at an effector:target ratio of 10:1 in IFNγ ELISpot plates. Plates were developed 48 hrs later and the number of spots counted. **C,D.** Following hydrodynamic injection of hMet + S45Y ß-Catenin (day 0), animals were treated starting on days 21,23,25,28,30,32 with anti-PD-L1 (200µg/injection) and with 10^7 pfu VSV-IFNß on days 38,40,42 (**C**) or with 10^7 pfu of VSV-SB-HCC1,2,3 on days 21,23,25 (**D**). CD8+ T cells were purified from the splenocytes and co-cultured with either a 1:1;1 mixture of live SB-HCC 1,2,3 explant cells as targets at an effector:target ratio of 10:1 (anti-SB-HCC CD8+ T cell response) or with 5µg/ml of the immunodominant VSV-N_{52-59} peptide to stimulate a recall response to VSV (anti-VSV-N_{52-59} CD8+ T cell response) in IFNγ ELISpot plates. Plates were developed 48 hrs later and the number of spots was counted. **E,F.** Following hydrodynamic injection of hMet + S45Y ß-Catenin (day 0), animals were treated starting on days 21,23,25,28,30,32 with anti-PD-L1 (200µg/injection) and with 10^7 pfu VSV-SB-HCC1,2,3 (**E**) or with 10^7 pfu of VSV-ASMEL (**F**) on days 38,40,42. CD8+ T cells were purified from the splenocytes and co-cultured with either a 1:1;1 mixture of live SB-HCC 1,2,3 explant cells as targets at an effector:target ratio of 10:1 (anti-SB-HCC CD8+ T cell response); with live B16 melanoma cells (anti-B16 CD8+ T cell response) or with 5µg/ml of the immunodominant VSV-N_{52-59} peptide to stimulate a recall response to VSV (anti-VSV-N_{52-59} CD8+ T cell response) in IFNγ ELISpot plates. Plates were developed 48 hrs later and the number of spots was counted. For all experiments, spleens from 3 mice per group per timepoint were harvested on days 28,49 and either day 90 post hydrodynamic injection or at terminal endpoint.

Fig. S8A

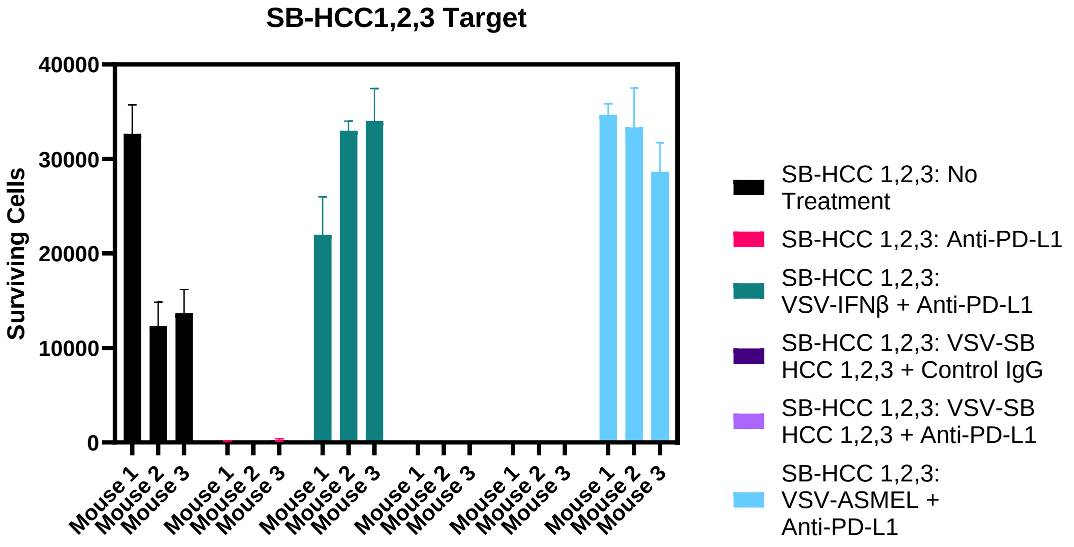

Fig. S8B

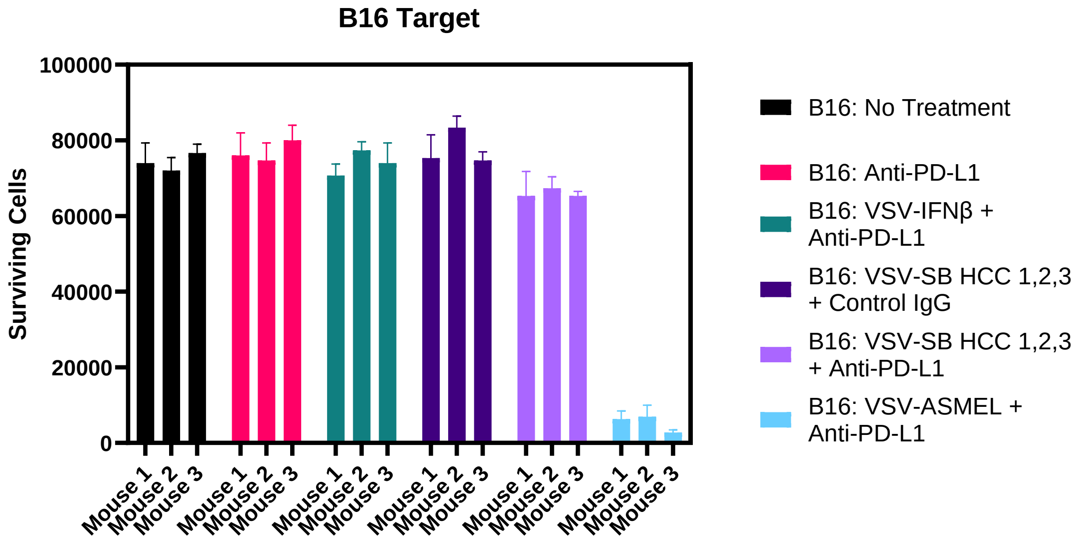

Supplemental Figure 8. CD8+ T cells induced by both anti-PD-L1 ICI and by VSV-SB-HCC1,2,3 are lytic to SB-HCC tumor cells.

CD8+ T cells harvested at day 49 post hydrodynamic injection from each mouse in the treatment groups of **Supplemental Figure 6 A-F** were co-cultured with a 1:1:1 mixture of 10^4 live SB-HCC 1,2,3 explant cells (anti-SB-HCC CD8+ T cell response), or with 10^4 live B16 melanoma cells (anti-B16 CD8+ T cell response), as targets at an effector:target ratio of 10:1. Both target populations were incubated overnight in IFNγ to induce MHC Class I expression, washed three times with PBS, and then co-cultured with the T cells. 72 hours later wells were washed three times with PBS to remove non adherent T cells and dead cells and the number of surviving cells were counted. Technical triplicates were performed for each sample.

Fig. S9

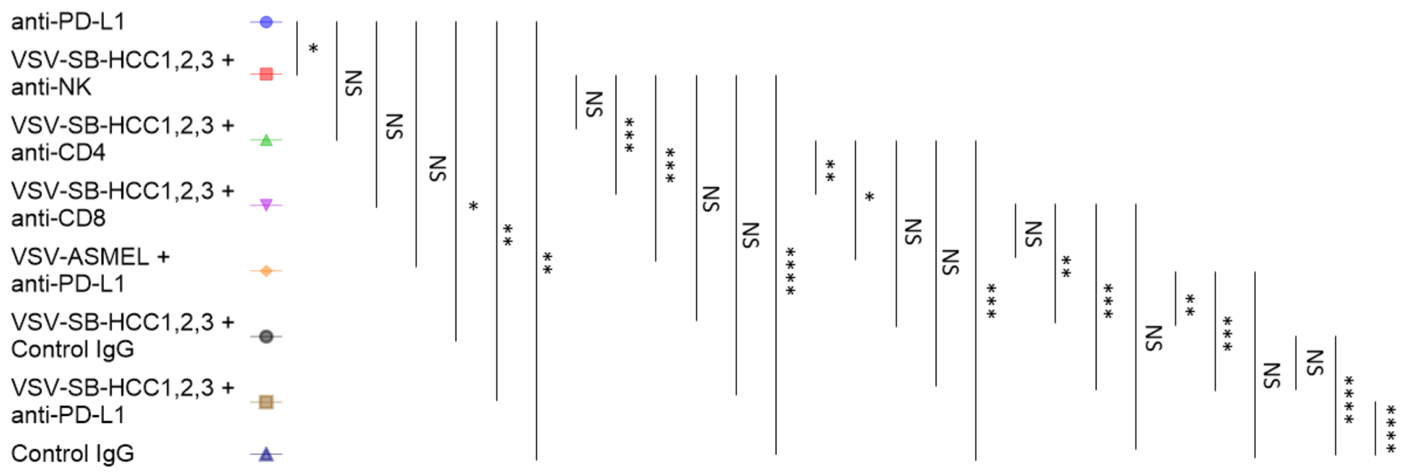
anti-PD-L1
VSV-SB-HCC1,2,3 + anti-NK
VSV-SB-HCC1,2,3 + anti-CD4
VSV-SB-HCC1,2,3 + anti-CD8
VSV-ASMEL + anti-PD-L1
VSV-SB-HCC1,2,3 + Control IgG
VSV-SB-HCC1,2,3 + anti-PD-L1
Control IgG

Supplemental Figure 9. Significance values of different treatment groups of Figure 7F.
