# Supplementary material for: Expression of tumor antigens within an oncolytic virus enhances the anti-tumor T cell response
Source: Nat Commun. 2024 Jun 27;15:5442. doi: 10.1038/s41467-024-49286-x (PMC11211353; doi:10.1038/s41467-024-49286-x)
Supplement: Supplementary file 3 — Reporting Summary [file 41467_2024_49286_MOESM3_ESM.pdf]

nature portfolio

Corresponding author(s): DAPR NCOMMS-23-53622B

Last updated by author(s): 2024-May 22

Reporting Summary

Nature Portfolio wishes to improve the reproducibility of the work that we publish. This form provides structure for consistency and transparency in reporting. For further information on Nature Portfolio policies, see our Editorial Policies and the Editorial Policy Checklist.

Statistics

For all statistical analyses, confirm that the following items are present in the figure legend, table legend, main text, or Methods section.

n/a	Confirmed	
☐	☒	The exact sample size (*n*) for each experimental group/condition, given as a discrete number and unit of measurement
☐	☒	A statement on whether measurements were taken from distinct samples or whether the same sample was measured repeatedly
☐	☒	The statistical test(s) used AND whether they are one- or two-sided<br>*Only common tests should be described solely by name; describe more complex techniques in the Methods section.*
☐	☒	A description of all covariates tested
☐	☒	A description of any assumptions or corrections, such as tests of normality and adjustment for multiple comparisons
☐	☒	A full description of the statistical parameters including central tendency (e.g. means) or other basic estimates (e.g. regression coefficient) AND variation (e.g. standard deviation) or associated estimates of uncertainty (e.g. confidence intervals)
☐	☒	For null hypothesis testing, the test statistic (e.g. *F*, *t*, *r*) with confidence intervals, effect sizes, degrees of freedom and *P* value noted<br>*Give P values as exact values whenever suitable.*
☒	☐	For Bayesian analysis, information on the choice of priors and Markov chain Monte Carlo settings
☒	☐	For hierarchical and complex designs, identification of the appropriate level for tests and full reporting of outcomes
☒	☐	Estimates of effect sizes (e.g. Cohen's *d*, Pearson's *r*), indicating how they were calculated

Our web collection on statistics for biologists contains articles on many of the points above.

Software and code

Policy information about availability of computer code

Data collection	No custom code was used. Data was collected using FlowJo 10 and NET MHC 2.0 binding affinity algorithm. http://www.cbs.dtu.dk./services/NetMHC
Data analysis	No custom code was used for analysis. Analysis was conducted with GraphPad Prism 10 and tsne/Shiny through Cytokit

For manuscripts utilizing custom algorithms or software that are central to the research but not yet described in published literature, software must be made available to editors and reviewers. We strongly encourage code deposition in a community repository (e.g. GitHub). See the Nature Portfolio guidelines for submitting code & software for further information.

Data

Policy information about availability of data

All manuscripts must include a data availability statement. This statement should provide the following information, where applicable:

- Accession codes, unique identifiers, or web links for publicly available datasets
- A description of any restrictions on data availability
- For clinical datasets or third party data, please ensure that the statement adheres to our policy

The data generated in this study have been deposited in a publically available figshare database, https://doi.org/10.6084/m9.figshare.25651704.v1

Research involving human participants, their data, or biological material

Policy information about studies with human participants or human data. See also policy information about sex, gender (identity/presentation), and sexual orientation and race, ethnicity and racism.

Reporting on sex and gender	N/A
Reporting on race, ethnicity, or other socially relevant groupings	N/A
Population characteristics	N/A
Recruitment	N/A
Ethics oversight	N/A

Note that full information on the approval of the study protocol must also be provided in the manuscript.

Field-specific reporting

Please select the one below that is the best fit for your research. If you are not sure, read the appropriate sections before making your selection.

☒ Life sciences ☐ Behavioural & social sciences ☐ Ecological, evolutionary & environmental sciences

For a reference copy of the document with all sections, see nature.com/documents/nr-reporting-summary-flat.pdf

Life sciences study design

All studies must disclose on these points even when the disclosure is negative.

Sample size	All experiments were designed, powered and analyzed by consultation with the Mayo Clinic Bioinformatics and Statistical Core. Many studies included "control" groups important to verify that the animal study was performed correctly, but are not of biological interest. For all analyses, α=0.05 were used. Assuming σ=30, 10 mice/grp will provide 80% power to detect a difference of 40d, with a two-sided α=0.05. Alternatively, assuming σ=6 we would be able to detect a difference of 10d. Large effect sizes were expected and we have been able to detect statistically-significant changes with 7-8/group, highlighting the feasibility of these studies.
Data exclusions	None
Replication	All experiments replicated at least once
Randomization	For in vivo studies, mice were randomized at time of tumor implantation using the GraphPad QuickCalcs online tool (https://www.graphpad.com/quickcalcs/randMenu/). For in vitro studies, no randomization was performed as cell used in this study were pulled from a single preparation with no reason to believe that the spacial location in the well impacted results.
Blinding	Mice were assessed by a single blinded individual. For in vitro studies The investigators were not blinded to the allocation of groups during experiments or subsequently during the analysis. Fully blinded experiments were not possible due to personnel availability to accommodate such situations.

Reporting for specific materials, systems and methods

We require information from authors about some types of materials, experimental systems and methods used in many studies. Here, indicate whether each material, system or method listed is relevant to your study. If you are not sure if a list item applies to your research, read the appropriate section before selecting a response.

Materials & experimental systems

n/a	Involved in the study	
☐	☒	Antibodies
☐	☒	Eukaryotic cell lines
☒	☐	Palaeontology and archaeology
☐	☒	Animals and other organisms
☒	☐	Clinical data
☒	☐	Dual use research of concern
☒	☐	Plants

Methods

n/a	Involved in the study	
☒	☐	ChIP-seq
☐	☒	Flow cytometry
☒	☐	MRI-based neuroimaging

Antibodies

Antibodies used	CD3 (Biolegend # 100236 clone 145-2C11, dilution 1:500), CD8a (Biolegend #100738/100747, clone 53-6.7, dilution 1:1000), CD4 (Biolegend #100451 clone GK1.5), PD1 (Biolegend # 109110, clone RMP1-30, dilution 1:200),TIM3 (Biolegend #119704, clone RMT3-23, dilution 1:200), CD11c (BD Biosciences clone HL3), I-A/I-E (MHCII) (Biolegend M5/114.15.2), CD86 (Biolegend #105037, clone GL-1), and fixable live dead viability dye (Zombie NIR). Cells were stained with the H-2Kb VSV NP52–59 RGYVYQGL (Brilliant Violet 421–labeled) tetramer at a dilution of 1:500 or the H-2Kb chicken ova257–264 SIINFEKL (APC-labeled) tetramer at a dilution of 1:150, which were obtained from the National Institutes of Health Tetramer Core Facility.
Validation	All antibodies used were validated by the manufacturer, antibody-specific staining was compared to FMO samples. Anti-mouse CD3 was validated using C57BL/6 mouse splenocytes https://www.biolegend.com/en-us/products/apc-anti-mouse-cd3-antibody-8055<br>Anti-mouse CD8 alpha was validated using C57BL/6 mouse splenocytes https://www.biolegend.com/en-us/products/brilliantviolet-421-anti-mouse-cd8a-antibody-7138<br>Anti-mouse CD4 was validated using C57BL/6 mouse splenocytes https://www.biolegend.com/en-us/products/brilliant-violet-605-anti-mouse-cd4-antibody-10708<br>Anti-mouse PD-1 was validated using Con A-stimulated (day-3) Balb/c mouse splenocytes https://www.biolegend.com/en-us/products/pe-cyanine7-anti-mouse-cd279-pd-1-antibody-3612<br>Anti-TIM-3 was validated using Mouse Tim-3 transfected cells<br>https://www.biolegend.com/en-us/products/pe-anti-mouse-cd366-tim-3-antibody-2657<br>Anti-mouse CD11c was validated using C57BL/6 mouse splenocytes https://www.bdbiosciences.com/en-us/products/reagents/flow-cytometry-reagents/research-reagents/single-color-antibodies-ruo/apc-hamster-anti-mouse-cd11c.550261<br>Anti-mouse I-A/I-E was validated using C57BL/6 mouse splenocytes https://www.biolegend.com/de-at/products/fitc-anti-mouse-i-a-i-e-antibody-366<br>Anti-mouse CD86 was validated using C57BL/6 mouse splenocytes<br>https://www.biolegend.com/en-ie/products/brilliant-violet-605-anti-mouse-cd86-antibody-7798?GroupID=BLG11928

Eukaryotic cell lines

Policy information about cell lines and Sex and Gender in Research

Cell line source(s)	BHK cells were obtained from the ATCC
Authentication	Authenticated by commercial source, otherwise not authenticated.
Mycoplasma contamination	Cell lines were routinely tested for mycoplasma and found to be negative.
Commonly misidentified lines (See ICLAC register)	No commonly misidentified cell lines were used in this study.

Animals and other research organisms

Policy information about studies involving animals; ARRIVE guidelines recommended for reporting animal research, and Sex and Gender in Research

Laboratory animals	C57Bl/6, FVB mice all 6-8 weeks of age
Wild animals	None
Reporting on sex	Yes, in manuscript
Field-collected samples	No field collected samples were used in this study
Ethics oversight	Yes, Institutional Animal Care and Use Committee at Mayo Clinic

Note that full information on the approval of the study protocol must also be provided in the manuscript.

Flow Cytometry

Plots

Confirm that:

☒ The axis labels state the marker and fluorochrome used (e.g. CD4-FITC).

☒ The axis scales are clearly visible. Include numbers along axes only for bottom left plot of group (a 'group' is an analysis of identical markers).

☒ All plots are contour plots with outliers or pseudocolor plots.

☒ A numerical value for number of cells or percentage (with statistics) is provided.

Methodology

Sample preparation	Described in Methods of manuscript
Instrument	Bio-Rad ZE5 Cell Analyzer
Software	FlowJo 10.10
Cell population abundance	Varies, at least 10,000 per 4th level population (CD8/CD4/CD11c)
Gating strategy	FSC, SSC, Live/Dead, CD11c/IAIE/CD8/CD4, TIM3/PD1/PDL1/CD86/SIIN/VSV-N

☒ Tick this box to confirm that a figure exemplifying the gating strategy is provided in the Supplementary Information.
